# Piecing the puzzle together: a revisit to transcript reconstruction problem in RNA-seq

**DOI:** 10.1186/1471-2105-15-S9-S3

**Published:** 2014-09-10

**Authors:** Yan Huang, Yin Hu, Jinze Liu

**Affiliations:** 1 Department of Computer Science, University of Kentucky, Lexington, KY, USA

**Keywords:** Transcript reconstruction, Transcript quantification, Transcriptome, RNA-seq

## Abstract

The advancement of RNA sequencing (RNA-seq) has provided an unprecedented opportunity to assess both the diversity and quantity of transcript isoforms in an mRNA transcriptome. In this paper, we revisit the computational problem of transcript reconstruction and quantification. Unlike existing methods which focus on how to explain the exons and splice variants detected by the reads with a set of isoforms, we aim at reconstructing transcripts by piecing the reads into individual *effective *transcript copies. Simultaneously, the quantity of each isoform is explicitly measured by the number of assembled effective copies, instead of estimated solely based on the collective read count. We have developed a novel method named *Astroid *that solves the problem of effective copy reconstruction on the basis of a flow network. The RNA-seq reads are represented as vertices in the flow network and are connected by weighted edges that evaluate the likelihood of two reads originating from the same effective copy. A maximum likelihood set of transcript copies is then reconstructed by solving a minimum-cost flow problem on the flow network. Simulation studies on the human transcriptome have demonstrated the superior sensitivity and specificity of Astroid in transcript reconstruction as well as improved accuracy in transcript quantification over several existing approaches. The application of Astroid on two real RNA-seq datasets has further demonstrated its accuracy through high correlation between the estimated isoform abundance and the qRT-PCR validations.

## Background

The advent of RNA-seq technologies has made it possible to characterize the mRNA transcriptome of a cell through massively parallel sequencing. A typical RNA-seq protocol works by randomly fragmenting the mRNA transcripts followed by sequencing a sample of the total fragments. The central problem of RNA-seq analysis is to recapitulate the variety and the abundance of the transcript isoforms from the sequenced short reads.

Many methods have been developed for transcript reconstruction and/or quantification recently. These methods include but are not limited to Cufflinks [1], Scripture [2], IsoLasso [3], RSEM [4], SLIDE [5], iReckon [6], MITIE [7], MultiSplice [8], *etc*. As pointed out by Behr *et al*. [7], it is important to perform isoform reconstruction and quantification simultaneously in order to maximize the performance of both steps. Although some of the existing methods claimed to conduct transcript reconstruction and quantification simultaneously [6-8], they still follow a two-step approach: 1) construct a set of candidate isoforms; 2) estimate the abundance of these isoforms by assigning reads probabilistically to each isoform through an optimization process. The relative abundance is calculated as FPKM, *i.e*., Fragments Per Kilobase of transcript per Million mapped reads, where the total number of fragments aligned to a transcript is averaged by the length of the transcript, regardless whether the reads are distributed evenly along the transcript.

**Figure 1:**
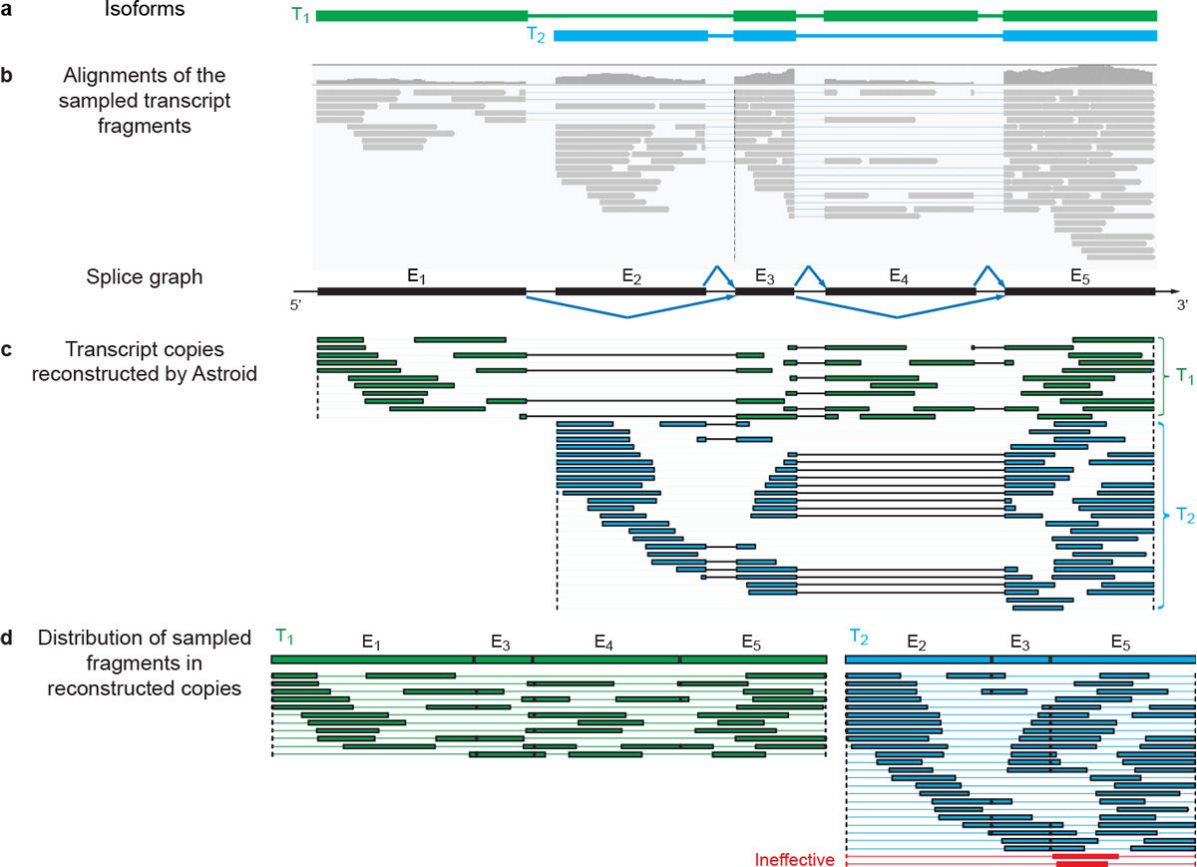
**Reconstruction of effective transcript copies by Astroid**. **(a) **The two isoforms from which transcript fragments are randomly sampled. **(b) **The alignments of the sampled fragments, plotted with IGV [36]. A splice graph can be built based upon the exons and splice junctions identified from the fragment alignments. **(c) **Effective transcript copies assembled by Astroid. Astroid successfully reconstructs the two expressed isoforms with no false positive. **(d) **The distribution of fragments in the effective copies. The likelihood of each copy is assessed according to the sizes of the fragments in the copy together with the between-fragment distances. Effective transcript copies will be identified and used to measure the abundance of each isoform. Note that this example shows only transcript fragments rather than the RNA-seq reads for simplified illustration. However, our method does take paired-end reads as input.

In this paper, we revisit the problem of transcript reconstruction. Instead of assigning reads probabilistically to a set of isoforms, we go one step further by answering how well individual reads may be pieced together to build copies of individual transcripts. We directly reconstruct *effective *transcript copies, each of which corresponds to a chain of non-overlapping transcript fragments (Figure 1). The contribution of each effective copy to the abundance of the corresponding isoform does not solely depend on the number of reads observed, but also on how consistent the distribution of the observed fragments is as compared to the expected process of mRNA fragmentation and sampling. This procedure allows us to explicitly take into account of the positional relationships among reads, which were generally ignored by existing methods. In the meantime, the total number of transcript copies constructed can be used to assess the transcript abundance. Therefore, we introduce a new measure for transcript quantification, namely*eTPM*, **e**ffective Transcripts Per Million. The eTPM of an isoform *i *is calculated as:

(1) $$\text{eTP}{\text{M}}_{i}=\frac{\text{e}{\text{T}}_{i}\times 10^{6}}{\Sigma_{j}\text{e}{\text{T}}_{j}}$$

where eT*_i _*is the number of effective copies of isoform *i *and Σ*_j _*eT*_j _*accounts for the total number of transcripts in the transcriptome. With our approach, not only do the constructed effective copies convey the information of the exon composition of the transcript, but the number of copies also delivers an estimation of the relative abundance of each isoform. It is therefore truly *simultaneous *in terms of transcript identification and quantification.

To this end, we have developed a novel computational algorithm *Astroid *(Transcript reconstruction through **as**sembly of effective **tr**anscript c**o**pies guided by the fragment **d**istance.). We model the relation of all observed reads using a directed flow network, with reads connected by edges whose weight represents the likelihood that two reads may coexist in a transcript. The most likely set of transcript copies is reached by solving a min-cost flow problem given the flow network. A compression scheme is developed to speed up the performance for genes with high read coverage. The model is further consolidated by adding Multi-Splice features [8], reads that span multiple alternative splicing events, to avoid the identification of spurious transcripts.

We have compared the performance of our method with a number of state-of-the-art methods including Cufflinks [1], Scripture [2], IsoLasso [3] and Trinity [9]. Simulation studies on the human transcriptome datasets have demonstrated Astroid's superior sensitivity and precision on transcript discovery. The eTPM estimate calculated from the number of effective transcript copies assembled by Astroid has exhibited an improved correlation with true transcript abundance than FPKM estimates. The evaluations on the MAQC human brain dataset and the Alexa-seq dataset further demonstrated the effectiveness of our method in real applications, in which Astroid provided slightly more consistent estimates for transcript abundance with qRT-PCR validations than other methods.

## Effective transcripts

We propose to construct a set of *effective transcript copies *which simultaneously explain the observed reads and estimate the transcript abundance.

The sampled fragments typically do not immediately follow each other and fragments may not be sampled immediately at the start/end of a transcript. We model the positional relationship among the fragments by considering the *size distribution *of 1) the fragments, 2) the gap between two adjacent fragments, and 3) the gap from the transcription start site to the first fragment and from the last fragment to the transcription termination site. We use $R_{t}^{fr}$ to denote the set of transcript fragments in a copy *t*. Each fragment can be identified by a mate pair of reads. The set of betweenfragment gaps in *t *is denoted as $R_{t}^{gap}$.

The likelihood of the transcript *t *is then interpreted as the joint likelihood of all its fragments together with the gaps according to their sizes. To simplify the model, we assume that the sizes of the fragments and the gaps follow the same distribution, whose density function is denoted as *d*(*·*).

Let isoform *i *be the isoform that *t *is copied from, denoted as *t *∈ *i*. Because different copies may have different number of component fragments, we use the geometric mean of the probabilities of all fragments and gaps in *t *to evaluate the likelihood of *t*,

(2) $$L\left(t\right)={\left(\underset{r\in R_{t}^{gap}}{\prod }d\left(len\left(r\right)\right)\underset{r\in R_{t}^{fr}}{\prod }d\left(len\left(r\right)\right)\right)}^{\frac{1}{\left|R_{t}^{gap}|+|R_{t}^{fr}\right|}}$$

Generally, L(t) represents a central tendency of the probability of the fragments and gaps contained in *t*. It is possible to model the size distribution of the gaps differently, with more complex distributions. However, as the experimental results have suggested, the approximation in our simplified model is sufficient. The distribution of *d*(*·*) will be discussed later in this section.

We further determine the effectiveness of a copy *t *by assessing the probability of observing a copy with likelihood no greater than L(t).

**Definition **For a transcript *t*, let I(t) denote the subdomain of the size density *d*(*·*) such that ∀*_x _*∈ I(t), *d*(*x*) *≤ *L(t). Then *t *is **effective **if the cumulative density integrated over all *x *∈ I(t) is no greater than a significance level *τ*, i.e., $\int_{I_{t}}d\left(x\right)dx\;\leq \tau$, where *τ *controls the probability of falsely considering *t *as ineffective.

As a convention in hypothesis testing, *τ *is often set as 0.05. For a given *τ*, the set of all effective transcript copies of isoform *i *is denoted as: $S_{i}=\left\{t:\;\int_{I_{t}}d\left(x\right)dx\;\leq \tau \right\}$. Then the abundance of isoform *i *is measured by the number of its effective copies, called *effective transcripts *(eT), eT*_i _*= *|S_i_|*.

Under the assumption of a uniformly random fragmentation process, the size distribution of the fragments generated from isoform *i *can be approximated as a characterized Weibull distribution [10,11] with two parameters *δ_i _*and *η*. The isoform-specific shape parameter *δ_i _*depends on the logarithm of the molecule length of *i*, and the scale parameter *η *reflects the fragmentation intensity which is constant across all transcripts in one experiment [10]. In this paper, the distribution of *d*(*·*) is approximated using the Weibull distribution, *d*(*·*) = *PW *(*·|δ_i_, η*).

## Effective transcripts per million (eTPM)

We define the relative expression estimate *effective transcripts per million *of isoform *i *by normalizing eT*_i _*by the total effective transcript copies in the transcriptome (Equation 1).

There exist two other measurements focusing on quantifying the relative isoform expression levels. They are both based on the number of reads on the isoform. One is FPKM [1]. For an isoform *i*, it approximates the transcript abundance by normalizing the number of fragments on the isoform *N_i _*by the isoform length *len*(*i*), and uses the total number of fragments per million as a measure of total transcripts in the transcriptome (Equation 3). However, when comparing the isoform abundance among samples, the latter approximation is not accurate due to the variant size distribution of the transcripts among different samples [12].

(3) $$\text{FPK}{\text{M}}_{i}=\frac{N_{i}}{\frac{len\left(i\right)}{10^{3}}\cdot \frac{N}{10^{6}}}=\frac{N_{i}\cdot 10^{9}}{len\left(i\right)}$$

Another measure TPM [4,12], *i.e*., Transcripts Per Million, resolves the inconsistency problem. It approximates the transcript number by normalizing the cumulative per base read coverage by the isoform length. TPM of an isoform *i *is then calculated as in Equation 4 with summing up the estimated abundance of all isoforms accounting for the total number of transcripts in the transcriptome.

(4) $$\text{TP}{\text{M}}_{i}=\frac{\frac{N_{i}len\left(r\right)}{len\left(i\right)}\cdot 10^{6}}{\sum_{j}\frac{N_{j}len\left(r\right)}{len\left(j\right)}}=\frac{N_{i}len\left(r\right)\cdot 10^{6}}{len\left(i\right)\cdot \sum_{j}\frac{N_{j}len\left(r\right)}{len\left(j\right)}}$$

Here *len*(*r*) refers to the expected fragment length.

However, it is unclear how well *N_i _× *10^3^*/len*(*i*) in FPKM and *N_i_len*(*r*)*/len*(*i*) in TPM can approximate the true abundance of one isoform because it is impossible that all observed fragments can be tightly arranged one after the other (Figure 1b) making every single base of the isoform covered by the read.

Unlike FPKM or TPM, eTPM explicitly considers the possible gaps between two adjacent fragments on the same transcript copy. Since eTPM is normalized by the total number of transcripts in a sample, it can be invariant across samples [12]. While both FPKM and TPM treat each read independently and consider them as the same, the effective transcripts used in eTPM is assessed according to the distribution of their fragments. In real experiments, the position distribution of sampled fragments may not be uniform due to PCR amplification error [13] or sampling biases [14-16]. The affected reads will form ill transcripts copies with only small fractions sampled. These ill transcripts will be recognized during the eTPM calculation, which allows for a more robust abundance measure (Figure 1d).

Although this measurement relies on the quantity of assembled effective copies rather than the number of reads, it is derived based on the same assumption as the other measurements regarding the abundance. Longer transcripts require more reads to construct an effective copy. Hence eTPM of different transcripts can be compared directly without the normalization by transcript length.

## Method

The assembly of effective transcript copies with RNA-seq reads is achieved by solving a minimum-cost flow problem. In this section, we detail the modeling of the problem, its solution and various improvements over the basic approach. The input to our method is the genomic alignments of the paired-end reads to the reference genome [17,18]. Another important data structure we used is the splice graph [19-22] (Figure 1b). The splice graph is constructed directly from the read alignments using the method described by Hu *et al*. [20], and will be used to infer potential transcripts where a pair of reads come from. In general, the exons are identified as the genomic regions covered by abundant reads. These exons constitute the vertices of the splice graph. The spliced read alignments contain splice junctions, each of which spans a pair of exons. The splice junctions make the edges in the splice graph, whose directions can be defined by the direction of the transcription. In addition, the donor and acceptor sites of a splice junction also determine the boundaries of an exon. A path in the splice graph corresponds to (part of) a possible isoform.

### Read flow network

We model the relationships among reads using a flow network, namely the Read Flow Network *RF N *= 〈*V, E, W, source, sink*〉. The vertex set *V *corresponds to the union of the set of reads and the set of transcription start/termination sites (The transcription start sites and termination sites can be either inferred as the genomic positions that exhibit certain characteristic signatures [23,24] or provided from existing transcript annotation). There are two types of edges between two read vertices, the *in-fragment *edges and the *between fragment *edges. The in-fragment edges (denoted as *E^in^*) refer to edges between reads generated from the same fragment. In the case of paired-end reads, the edge is between the two mates of a paired read. The between-fragment edges (denoted as *E^btwn^*) refer to the edges that connect one fragment with its downstream fragment. In this case, an edge usually connects the 3' end read of a fragment (or a transcription start site) to the 5' end of a downstream fragment (or a transcription termination site). Let ϕ(*v*) be the exon in the splice graph where a read *v *is aligned to. For two vertices *v*_1_*, v*_2 _∈ *V*, There exists an edge between *v*_1_

and *v*_2 _for each unique path *ρi*(ϕ(*v*_1_)*, ϕ*(*v*_2_)) between exon ϕ(*v*_1_) and exon ϕ(*v*_2_) in the splice graph. In presence of alternative splicing, there may exist more than one paths in the splice graph from *ϕ*(*v*_1_) to ϕ(*v*_2_). In this case, multiple edges may be added to include all paths.

The weight of an edge *e, e *∈ *E *corresponding to a path *ρ_i_*(ϕ(*v*_1_)*, ϕ*(*v*_2_)) between two reads *v*_1 _and *v*_2_, reflects the likelihood of the two reads coming consecutively from the same transcript copy. It is evaluated by the probability of observing a portion between *v*_1 _and *v*_2 _on path *ρ_i_*(ϕ(*v*_1_)*, ϕ*(*v*_2_)). For *e *∈ *E^in ^*and *e *∈ *E^btwn^, len*(*e*) denotes the size of the observed fragment and size of the between-fragment gap, respectively. Assuming both sizes follow a Weibull distribution, then the probability of *e *is calculated as *P *(*e*) = *PW *(*len*(*e*)). The weight of *e *in *RF N *is defined as the negative logarithm of its likelihood, *w*(*e*) = *− *log *P *(*e*).

Lastly, the network *RF N *is augmented by adding a virtual *source *and a virtual *sink *to initiate and terminate all transcript copies. Directed edges will be built from *source *to all vertices that correspond to transcription start sites and from all vertices that correspond to transcription termination sites to *sink*. Moreover, one edge is added to connect *source *to *sink*. The weights on these edges are always set as 0. Because every read may only originate from one transcript copy, the capacity constraint on every vertex that represents a read is set as 1. The capacities on *source, sink *and vertices that represent transcription start/termination sites all equal to the number of vertices that represent the reads.

**Figure 2:**
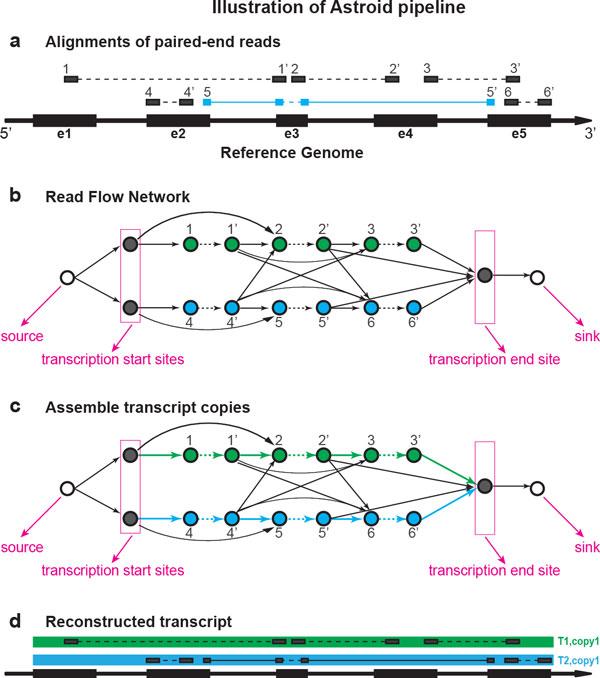
**(a) Alignments of the sequenced paired-end RNA-seq reads on the reference genome**. **(b) **The read flow network that relates reads with in-fragment edges (dashed arrows) and between-fragment edges (solid arrows). **(c) **Solve a minimum flow (colored) on the read flow network. **(d) **The assembled effective transcript copies with maximized likelihood.

In this way, each transcript copy can be represented as a *source *to *sink *path (flow) (Figure 2b). Let  T denote one set of transcript copies in *RF N*. For every copy *t *∈  T, the likelihood of *t *can be evaluated as the product of the probabilities of its reads (vertices included in *t*), the probabilities of its distances connecting paired-end reads (*in-fragment *edges in *t*) and the probabilities of its distances connecting transcript fragments (*between-fragment *edges in *t*). The transcript copies in  T are considered mutually independent because the vertices and edges included in one copy are exclusive. Hence, the likelihood of  T can be written as the joint probability of all the transcript copies in  T,

(5) $$\begin{array}{cc} P\left(T\right) & =\underset{t\in T}{\prod }P\left(t\right)\\ & =\underset{t\in T}{\prod }\underset{v\in t\cap V}{\prod }P\left(v\right)\underset{e\in t\cap E^{in}}{\prod }P\left(e\right)\underset{e\in t\cap E^{btwn}}{\prod }P\left(e\right) \end{array}$$

The probability of a read *P *(*v*) can be calculated by considering the quality of its alignment quality [4]. The probability of an edge *P *(*e*) has been defined as *P_W _*(*len*(*e*) = *l|e*). Then the maximum likelihood set of transcript copies can be written as,

(6) $$\begin{array}{ccc} \hat{T} & =\text{arg}\underset{T}{\text{ max }}\text{log}P\left(T\right)=\text{arg}\underset{T}{\text{ max }}\underset{t\in T}{\sum }\left(\underset{v\in t\cap V}{\sum }\text{log}P\left(v\right)+\underset{e\in t\cap E^{in}}{\sum }\text{log}P\left(e\right)+\underset{e\in t\cap E^{btwn}}{\sum }\text{log}P\left(e\right)\right) & \\ & =\text{arg}\underset{T}{\text{ min }}\underset{t\in T}{\sum }\left(\underset{v\in t\cap V}{\sum }-\text{log}P\left(v\right)+\underset{e\in t\cap E^{in}}{\sum }-\text{log}P\left(e\right)+\underset{e\in t\cap E^{btwn}}{\sum }-\text{log}P\left(e\right)\right). \end{array}$$

Therefore, the problem of solving the maximum likelihood set of transcript copies is equivalent to a *minimum-cost flow *problem [25,26] on the flow network *RF N*,

(7) $$\hat{T}=\text{arg}\underset{f\left(\cdot \right)}{\text{min}}\underset{e\in E}{\sum }w\left(e\right)\cdot f\left(e\right),$$

where *f *(*e*) is the amount of flow on every edge *e*.

Generally, solving a minimum-cost flow problem requires the pre-knowledge of the amount of flow sending from source to sink, denoted as *k*. Here *k *is set as a comparably large value (e.g. the total number of reads), the edge connecting *source *to *sink *will consume the extra amount of flow beyond the number of transcript copies which flow through the read vertices.

### Acceleration with compressed flow network

**Figure 3:**
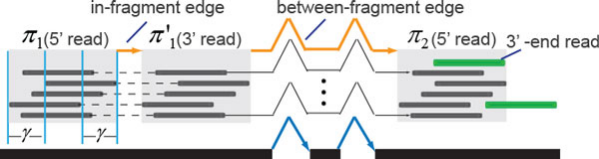
**An example of the compressed flow network**. Reads colored black are grouped into 3 clusters (light gray). Edges connecting the reads in the original RFN are collapsed into two edges (colored orange) in the compressed network. The two reads colored green cannot be clustered into *π*_2 _because they violate the vertex homogeneity and alignment adjacency, respectively.

The time complexity of the algorithms solving the minimum-cost flow problem is *O*(*|V |*^3^) [27,27,28], *|V | *is the number of vertices. Given the size of reads, which could be in the order of millions, the problem can be intractable. Here we introduce a heuristic to compress the read flow network into a much smaller network with minimal loss of accuracy. The idea is to remove highly repetitive reads in high coverage region by clustering these reads into groups while still retaining the relationships among them. Given a compression parameter *γ*, the vertex set *V *of the flow network can be partitioned into a set of clusters Π = {*π*_1_*, π*_2_*, · · ·, π_c_*} of *V*, such that the reads within each cluster contain consistent splice junctions; have homogenous outgoing edges and differ at most *γ *bases at both boundaries (Figure 3).

1 *Vertex homogeneity. ∀v*_1_*, v*_2 _∈ *π_i_, π_i _*∈ Π, *v*_1 _and *v*_2 _are either both the 5' end reads of one fragment or both the 3' end, and *v*_1 _and *v*_2 _either have the same set of splice junctions in their alignments or have no splice junctions;

2 *Edge homogeneity. ∀v*_1_, $v_{1}^{\prime }\in \pi_{i}$ ∀*v*_2_, $v_{2}^{\prime }\in \pi_{j}$, *π_i_, π_j _*∈ Π, there exists no edge between *v*_1 _and $v_{1}^{\prime }$ or between *v*_2 _and $v_{2}^{\prime }$, and the edges between *v*_1 _and *v*_2 _represent the same set of paths in the splice graph as the edges between $v_{1}^{\prime }$ and $v_{2}^{\prime }$;

3 *Alignment adjacency*. ∀*v*_1_*, v*_2 _∈ *π_i_, π_i _*∈ Π, the 5'-most base of *v*_1 _is at most *γ *bases away from that of *v*_2 _on the genome, and the same for their 3'-most bases.

In this way, the vertex set *V *can be reduced to the set of vertex clusters Π, and the duplicated edges between vertices of two clusters can be removed if they represent the same path in the splice graph. The distances of duplicated edges may differ by at most 2*γ *bases, but the minimum weight of all the duplicated edges will be assigned to the only edge kept in the compressed flow network. The capacity of each vertex changes to the size of the cluster, and the capacity of an edge is the number of duplicated edges in the original flow network. Therefore, *γ *adjusts the degree of heterogeneity of reads in each clusters. When *γ *goes larger, generally more reads on the same exon can be grouped into one cluster and more reads containing the same splice junctions can also be clustered together. As a result, the compressed flow network has less vertices and edges and its size will become closer to that of the splice graph. In practice, *γ *is set to half the expected fragment length, *γ *= *len*(*r*)*/*2, which improves speed by significantly reducing the size of the flow graph while retaining high accuracy by allowing sufficient overlaps among reads in a cluster.

The calculation of partition can finish in *O*(*|V |*) time. Our simulation studies have demonstrated that the compression may greatly reduce the time cost while maintaining a satisfactory accuracy of the assembled transcript copies.

### Consolidating transcript reconstruction across alternative splicing events

The alternative splicing events (ASEs) happening between two cluster vertices will lead to more than one ways to connect them. In presence of multiple ASEs, it is important to avoid a simple enumeration of all possible isoforms from the combinations of variants in the ASEs. Therefore, we leverage the reads that span multiple ASEs to help evaluate the likelihood of existence of a possible isoform, using the MultiSplice features developed in our previous work [8]. Formally, a MultiSplice is a sequence of adjacent exons on a path of the splice graph, such that reads longer than a particular length may span all these exons. These features are calculated and incorporated here to reduce the possibility of linking the vertices into false transcripts.

**Figure 4:**
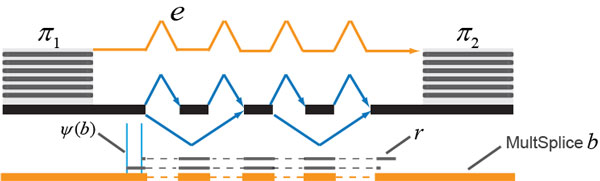
**An example of a MultiSplice feature**. Two ASEs (both are exon-skipping) reside between the clusters π1 and π2. The feature b consists of 5 exons on the path indicated by edge e. Two possible alignments of read r are shown in order for r to span b and confirm the existence of edge e. The possible positions of such alignments then give a sampling window of b (the window bounded by the two light blue lines).

Let *e *denote an edge in the compressed flow network. Let *b *denote the MultiSplice feature that consists of the same set of exons as the path indicated by *e*. Let *ψ*(*b*) denote the size of the sampling window of *b *[8], which is the number of positions that a read could fall on in order to cover all exons of *b *(Figure 4). If no read is observed spanning *b*, the existence of edge *e *cannot be confirmed. In this case we assign a penalty to the weight of *e *by calculating the probability of observing no spanning read,

$$P\left(e\;\text{not confirmed}\right)={\left(1-\frac{\psi \left(b\right)}{len\left(e\right)}\right)}^{\left|c_{e}\right|}$$

where *c_e _*is the capacity on *e*. If *ψ*(*b*) = 0, no read may span *b *at the given read length, *P *(*e *not confirmed) =

1. Thereafter, we adjust the weight of *e *by adding *-- *log *P *(*e *not confirmed) to *w*(*e*).

**Figure 5:**
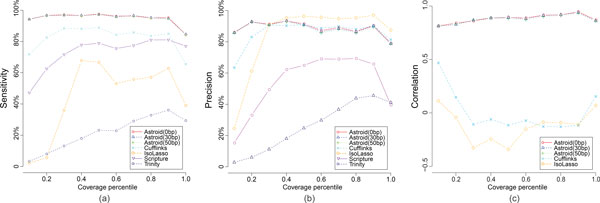
**Performance comparison of Astroid with 3 different compression parameters (0 bp, 30 bp and 50 bp), Cufflinks, IsoLasso, Scripture and Trinity on 30M 2*×*75 bp (insert size around 250 bp) paired-end dataset**. Evaluation measurements were plotted against different gene expression quantile (in 10% increments). (a) Each point in the plot represents the sensitivity of one method which is the ratio between the number of matched transcripts and the reference transcripts within one quantile. (b) Each point represents the precision of one method which is the number of matched transcripts and the total assembled transcripts within one quantile. (c) The correlation of transcript expression is computed on the set of matched transcripts for each method.

## Experimental results

We compared the performance of our method *Astroid *with four other state-of-the-art approaches for transcript reconstruction, including two "genome-guided" methods [29] with different heuristics, Cufflinks [1] and Scripture [2] (Cufflinks 2.0.2 and Scripture beta version 2 were downloaded, Cufflinks was run in the mode that carries out both reconstruction and quantification and without -g/-G option), one representative method for Lasso-based "genome-guided" assembly IsoLasso [3] (IsoLasso version 2.6.0) and one "genomeindependent" approach Trinity [9] (Trinity version 2012-10-05). The assembled transcripts from Trinity were generated in fasta format and were mapped to the reference genome using BLAT [30] with default parameters. Only hits with complete match were considered in the comparisons. To understand the sensitivity and specificity of the transcript reconstruction as well as the accuracy of transcript quantification, we first did comparison on all five approaches using simulated datasets of varying sampling depths. We then compared the genome-guided assembly methods (Trinity excluded) on two real RNA-seq datasets, MAQC data[31] and Alexa-seq data[32], where qPCR of a subset of transcripts are available to assess the accuracy of quantification using RNA-seq.

### Simulation study

**Data simulation**. We developed a simulator that mimics a real RNA-seq experiment and generates fragments from provided transcript copies. The simulation process consists of three steps: (1) Build a synthetic transcriptome by randomly assign copy numbers to all the genes and isoforms in the annotation database and set this as the true profiles. (2) Randomly cut the transcripts in the synthetic transcriptome into small fragments and dynamically check the lengths of the generated fragments. Fragments with lengths in a certain range (e.g. [150*bp*, 350*bp*]) are selected with probability to construct the sequencing library. This step stops when the number of fragments in the library exceeds the pre-specified sequencing depth. (3) 2*×*75*bp *paired-end reads are sampled from both ends of these selected fragments.

**Matching criteria**. We evaluate the assembly results using similar criteria proposed in IsoLasso [3]. The assembled transcripts are compared with all the expressed transcripts in the profile (referred as "reference transcripts"). Two multi-exon transcripts are considered matched if they satisfy that (1) they contain the same set of exons; (2) all the exon boundary coordinates are identical except the start of the first exon and the end of the last exon. Also, two single-exon transcripts match if and only if at least 50% of the exons are overlapped. We adopted sensitivity and precision to measure the accuracy of the assembly results. Let *M *denote the number of reference transcripts. *N *out of *M′ *assembled transcripts can be matched to the reference transcripts. Hence, $sensitivity=\frac{N}{M}$, and $precision=\frac{N}{M}$.

**Quantification accuracy criteria**. Both Cufflinks and IsoLasso quantify transcript expression in the unit of FPKM. In Astroid, we use eTPM. However, these measurements cannot be directly compared. Therefore, we evaluate the quantification accuracy by the correlation between the transcript abundances estimated by each method and the true profiles. Pearson correlation [33] is adopted for this assessment. Let *Y *denote the true copy numbers of the transcripts and *Ŷ *denote the estimated abundance. The correlation is calculated as *r*(*Y, Ŷ*) = *cov*(*Y, Ŷ*)*/*(*σ_Y _· σ_Ŷ_*), giving a value between *−*1 and +1. Higher correlation indicates more accurate estimation results.

**Results**. We conduct our first experiment to compare the performance of different methods on the transcriptome level. 30 million 2*×*75 bp paired-end reads (insert size around 250 bp) were simulated from the human transcriptome using RefSeq transcripts annotation. According to the profile, 18,374 transcripts from 13,030 genes were expressed. The reconstructed full-length transcripts of each method were matched against the ground truth, then the sensitivity and precision were assessed against different gene expression quantiles.

As shown in Figure 5(a), Astroid consistently acquired highest sensitivity with increasing gene coverage. Even for the lowly expressed genes (bottom 10%), Astroid successfully recovered around 95% of these transcripts which is more than at least 20% of all the other methods. The precision of Astroid also outperformed the others on the bottom genes (shown in Figure 5(b)). As gene expression climbs, the precisions became comparable between Astroid and Cufflinks, but were smaller than that of IsoLasso. This is probably related to the shrinkage strategy taken by IsoLasso which eliminates a large portion of transcripts through Lasso [34].

Figure 5(c) illustrates the quantification accuracy of each method. Astroid achieved highest correlation across different gene expression and demonstrated its ability of highly precise quantification through directly assembling transcript copies. However, both Cufflinks and IsoLasso showed very poor estimation. A further investigation on Cufflinks and IsoLasso abundance estimation results revealed that they both provided extremely high FPKM for short transcripts (less than 300 bp) which is quite inconsistent with the profile. Similar observation was also reported by Li, *et al*. [4]. Excluding the abnormal results on these short transcripts, the correlation increases for both methods, but still falls behind Astroid. Astroid, however, was not heavily affected by the length of transcripts because of its capability to explicitly model the distance between reads and transcription start and termination sites.

We also look into the effect of the compression parameter on Astroid. According to the results shown in Figure 5, we do observe that Astroid baseline (*γ *= 0*bp*) performs better than the other two with positive *γ*, but the difference is not that significant. Meanwhile, as shown in Supplementary Table 4, the time cost improves from 1 day to 1 hour as *γ *increases from 0 bp to 50 bp. This suggests that significant improved in efficiency can be achieved without much degradation of its performance. Therefore, in real practice, we may set the compression parameters at a comparably larger value (*γ *= *len*(*r*)*/*2). We use this setting in real data experiments.

We next evaluate how the sampling depth may affect the performance of each method. To do this, we first sample 10M and 20M 2*×*75 bp paired-end reads by random selection out of the 30M dataset. Table 1 shows the overall sensitivity, precision and correlation on these three datasets. From the statistics, we see that both the sensitivity and precision improve for all methods as more reads are sequenced. Apparently, higher sampling depth is more conducive for inferring transcript structures. Similar with previous observation, Astroid showed best performance against various sampling depths, which indicates that eTPM computed from the effective transcripts, is a robust measure for estimating the relative transcript abundance.

**Table 1:** Summary statistics of each method with various sampling depths.

Methods	sensitivity			precision			correlation (long transcripts only)		
	
	10M	20M	30M	10M	20M	30M	10M	20M	30M
Astroid(*γ *= 0)	79.28%	91.71%	94.30%	51.44%	80.23%	86.61%	.805 (.801)	.870(.868)	.922(.919)
Astroid(*γ *= 30)	79.20%	91.76%	94.22%	51.31%	80.01%	86.28%	.808(.805)	.872(.869)	.918(.914)
Astroid(*γ *= 50)	79.08%	91.64%	93.87%	51.18%	79.47%	85.78%	.808(.806)	.874(.870)	.912(.919)
Cufflinks	49.43%	74.48%	81.50%	51.31%	75.75%	79.55%	.106(.631)	-.033 (.773)	-.018(.808)
IsoLasso	2.86%	23.97%	45.83%	19.83%	75.26%	85.81%	-.027 (.356)	0.116 (.559)	.011 (.755)
Scripture	38.51%	62.13%	74.04%	12.46%	26.34%	39.74%	N/A	N/A	N/A
Trinity	3.36%	13.01%	23.04%	1.74%	6.13%	12.32%	N/A	N/A	N/A

Correlation values in parentheses are calculated on only long transcripts (length *>*300 bp, detailed counts in Supplementary Table 1.

### MAQC data study

For evaluation on real RNA-seq experiments, we first compared the four genome-guided transcript reconstruction approaches Astroid, Cufflinks, Scripture and IsoLasso using the RNA-seq dataset from Microarray Quality Control (MAQC) project Human Brain Reference (HBR) sample [31] (NCBI Short Read Archive accession number SRA012427). This dataset contained

23 million 2 ȕ 50 bp paired-end reads generated from three lanes. Besides RNA-seq data, 907 transcripts were analyzed with TaqMan qRT-PCR for their expression, including 893 that could be matched to RefSeq transcript annotation [35] (accession number GSE5350). We focused our analysis on this subset of validated transcripts.

**Figure 6:**
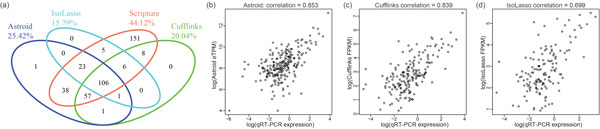
**(a)**. Venn Diagram of qRT-PCR validated transcripts reconstructed by Astroid, Cufflinks, Scripture and IsoLasso. (b)-(d) Scatter plots (on *log_e _*scale) of transcript abundance estimated by Astroid, Cufflinks and IsoLasso, respectively, against qRT-PCR expression on the set of qRT-PCR validated transcripts that are reconstructed in full length by each method.

Among the 893 qPCR-validated transcripts, Astroid correctly reconstructed 227, with a sensitivity of 25.42% (227 out of 893). This sensitivity is higher than those of Cufflinks (20.04% or 179 of 893) and IsoLasso (15.79% or 141 of 893). This demonstrates Astroid's good ability to reconstruct full-length transcripts. The venn diagram shown in Figure 6(a) illustrated a good consistency of their assembly results. We notice that Scripture reconstructed the most number of validated transcripts (44.12% or 394 of 893). This is due to the strategy of Scripture which tries to enumerate all possible transcripts given the exons and junctions observed from RNA-seq data. This strategy may highly increase the sensitivity but it also introduces large amount of false positives, especially on the genes with high coverage. In fact, the total number of assembled transcripts is 92,977 for Scripture which corresponds to a precision of 0.42% (394 of 92,977) and it is only half of Astroid (0.87% or 227 of 26,119). However, it is surprisingly that both Cufflinks and IsoLasso showed lower precision than Scripture on the identification of validated transcripts: 0.26% (179 of 69,011) and 0.10% (141 of 135,085). A close examination revealed that majority of their reconstructed transcripts are very short single-exon transcripts with low coverage, which are probably just background noises due to sequencing or mapping biases.

Next, we examined the transcript expression measured by qRT-PCR experiments and the expression estimated by each method (excluding Scripture) on the set of transcripts that were validated and correctly reconstructed: scatter plots are shown in Figure 6(b)(d). Transcript abundance inferred by Astroid reached a Pearson correlation as high as 0.853 on all the transcripts it correctly assembled, slightly higher than Cufflinks (0.839) and much higher than IsoLasso (0.699).

This result demonstrated that Astroid is competitive for transcript quantification. We further ran Cufflinks in its quantification-only mode by providing the RefSeq transcript annotation. The estimated transcript abundance by Cufflinks on all 893 validated transcripts had a Pearson correlation of 0.866, consistent with its previous reports on MAQC dataset for transcript quantification [15]. The difference between Cufflinks without transcript annotation and with annotation suggests that downstream analysis such as transcript quantification can be significantly altered by transcript reconstruction results. On the other hand, Astroid shows the prominent ability of discovering the underlying transcripts and providing reliable expression estimates simultaneously.

### Alexa-seq data study

We further applied Astroid and other methods to a real RNA-seq dataset used by Alexa-seq [32], an alternative expression/transcription analysis method. Total 262 million Illumina paired-end RNA-seq reads (36 bp or 42 bp) were generated from two cell lines: fluorouracil (5-FU)-resistant and -nonresistant human colorectal cancer cell lines, MIP101 and MIP/5-FU. The raw RNA-seq reads were downloaded from Alexa-seq website (http://www.alexaplatform.org/alexa_seq/). 167 million paired-end reads were generated from MIP101 sample and 89.82% of them were successfully mapped by MapSplice using human hg18 reference genome. The rest 95 million reads came from sample MIP/5-FU, among which 90.26% were mapped by MapSplice. Alexa-seq also provided qRT-PCR validation on 192 alternatively expressed exons. We focus the comparison of all the methods on identification of all the validated exons. One exon is considered reconstructed by one method if: (1) at least one assembled transcript contains this exon; (2) both boundaries of the identified exon have to match the hg18 annotation unless this exon is transcription start/end; (3) if the exon is transcription start/end, only downstream/upstream boundary of this exon is required to match the annotation, respectively. The estimated abundance on this exon is collected as the cumulative estimated abundance on the exon of all the transcripts assembled covering it.

Table 2 shows the number of validated exons successfully reconstructed and the correlation between the estimation and the qRT-PCR expression by each method. From the results, we observe that Astroid reconstructed the highest number of exons in both samples among all the assembly tools. This suggests that Astroid successfully reconstructed the transcripts containing these target exons. Meanwhile, correlation between estimated abundance and qRT-PCR expression was computed on the set of reconstructed exons by each method. Astroid and IsoLasso acquired the highest correlation (0.99) on sample MIP101, much higher than Cufflinks (*−*0.02). The correlation by Astroid dropped on MIP/5FU sample, but was still comparable to IsoLasso, which also outperformed Cufflinks.

**Table 2:** Summary statistics on the validated set of exons.

Methods	# exons reconstructed		correlation with qRT-PCR expression	
	
	MIP101	MIP/5-FU	MIP101	MIP/5-FU
Astroid	137	114	0.99	0.81
Cufflinks	124	66	*−*0.02	*−*0.03
IsoLasso	131	76	0.99	0.87
Scripture	105	60	N/A	N/A

Although Astroid consistently performs better than the other methods on the two real RNA-seq datasets, it is noticed that its improvement is not as significant as that in simulation experiments. After further investigation, we found that: (1) for real datasets, we only have access to a very small set of validated transcripts or exons supported by abundant read alignments. But for simulation, we sampled reads from the whole transcriptome containing genes with a large dynamic range in their expression. The splice junctions with relatively low read support tend to be filtered out by methods like Cufflinks and IsoLasso, which lead to their failure in reconstructing the correct set of full-length transcripts; (2) for MAQC dataset, the transcripts with PCR validation are mainly from single-isoform genes. As we know, it is easier to reconstruct and quantify single-isoform genes than multi-isoform genes. As a result, the differences among these methods are minimal.

## Discussion

In this article, we have proposed a novel method Astroid for simultaneous transcript reconstruction and quantification. Compared with existing methods which typically reconstruct isoforms in a splice graph, our approach provides a unique solution by piecing individual reads into a set of effective transcript copies. A novel measure for transcript abundance eTPM has also been defined based on the assembled effective copies, rather than indirect estimators that fully depend on the read count. The problem of the reconstruction of effective transcript copy has been modeled as a minimum-cost flow problem, which allows the solution of a maximumlikelihood set of copies.

We evaluated Astroid as well as four existing methods using both simulated data and real data. In general, the eTPM measure generated by Astroid has a better overall correlation with the ground truth or qRT-PCR measurement than FPKM output from Cufflinks and Isolasso. However, further validations using real datasets are still necessary in checking out the relationships among eTPM, TPM and FPKM in terms of their accuracy in inferring the abundance of alternative transcripts in multi-isoform genes as well as reconstructing isoforms of genes with relatively low expression. We are also interested in validating whether eTPM or TPM would be able to effectively normalize transcript abundance by the size of transcript library that is sample-specific, alleviating the risk of comparing transcriptoms with drastically different transcript composition.

We use the Weibull distribution to characterize the distribution of the fragment and between-fragment gap sizes. It was shown as an approximation of the fragment size distribution before size selection [10]. However, how to characterize the exact size distribution in real data needs further investigation. We are interested in answering the following questions: (1) would the Weibull distribution family fully capture the complexity of RNA-seq? (2) accordingly how would different distributions affect the assembly results?

Our approach is built on the assumption that short read sequencing may only capture a fraction of each mRNA molecule. Hence, the sampling "gaps" on transcripts that we have modeled has the potential to handle uneven read distribution due to various biases, such as positional bias and sequence bias. Ill-formed copies which contain only a proportion of the expected transcript may indicate an aberrant distribution of the observed reads and suggest possible biases. For example, if 3' end positional bias is observed, we may compensate the less sequenced 5' end by allowing a larger gap between 5' end and one fragment. We are currently working on potential methods to correct these biases within the existing framework.

## Availability

The software package can be accessed at http://www.netlab.uky.edu/p/bioinfo/Astroid. The program takes read alignment as input in SAM format, and provides reconstructed transcripts in the standard GTF format. The estimated abundance for each gene or isoform will be given in the units of eTPM.

## Competing interests

The authors declare that they have no competing interests.

## Authors' contributions

All authors developed the methodology and wrote the manuscript. Yan Huang developed the Astroid software package and carried out the experiments. All authors read and approved the final manuscript.

## Supplementary Material

Additional file 1 **Supplemental material**. List of notations used in the main manuscript and additional results on the simulated datasets from the whole human transcriptome. Click here for file

## Declarations

This work was supported by The National Science Foundation [CAREER award grant number 1054631 to J.L. and ABI/EF grant number 0850237 to J.L.]; National Institutes of Health [grant number P30CA177558 and 5R01HG006272-03 to J.L.].

This article has been published as part of BMC Bioinformatics Volume 15 Supplement 9, 2014: Proceedings of the Fourth Annual RECOMB Satellite Workshop on Massively Parallel Sequencing (RECOMB-Seq 2014). The full contents of the supplement are available online at http://www.biomedcentral.com/bmcbioinformatics/supplements/15/S9.
