# Supplementary material for: Piecing the puzzle together: a revisit to transcript reconstruction problem in RNA-seq
Source: BMC Bioinformatics. 2014 Sep 10;15(Suppl 9):S3. doi: 10.1186/1471-2105-15-S9-S3 (PMC4168703; doi:10.1186/1471-2105-15-S9-S3)
Supplement: Additional file 1 — Supplemental material. List of notations used in the main manuscript and additional results on the simulated datasets from the whole human transcriptome. [file 1471-2105-15-S9-S3-S1.pdf]

Supplemental Materials

The supplementary materia provides additional results on the simulated datasets from the whole human transcriptome.

Notations

Symbol	Meaning	Symbol	Meaning
i	isoform	$P_W(\cdot\|\delta_i, \eta)$	characterized Weibull distribution
t	transcript copy	$\phi(v)$	exon is splice graph a read v is mapped to
N_i	read count on isoform i	$\rho(\phi(v_1), \phi(v_2))$	a splice graph path between two exons
N	total read count in the transcriptome	$f(e)$	the amount of flow on edge e
$len(\cdot)$	length of $\cdot$ (read, transcript, etc.)	γ	compression parameter
R_t^{fr}	set of sampled fragments in copy t	π	read cluster
R_t^{gap}	set of between-fragment gap in copy t	b	MultiSplice feature
$d(\cdot)$	fragment/gap size distribution	$c(e)$	capacity on edge e in RFN
eT_i	effective transcripts of isoform i	$\psi(b)$	size of the sampling window of b

Table 1: Notations in the main manuscript.

Varying sampling depth

Table 2 includes statistics of the results on the 10M, 20M and 30M 2×75bp paired-end datasets. The mean fragment length of these datasets is 250bp. In the quantification accuracy analysis, both Cufflinks and IsoLasso show abnormally high FPKM on short transcripts. Therefore, a subset of transcripts with lengths great than 300bp are selected for Astroid, Cufflinks and IsoLasso for further assessment. The number of transcripts in this subset are shown in the last three columns.

Methods	# assembled genes			# assembled transcripts			# matched transcripts			# matched transcripts (long)		
	10M	20M	30M	10M	20M	30M	10M	20M	30M	10M	20M	30M
Astroid($\gamma = 0$)	22,327	14,679	13,548	28,321	21,002	20,006	14,567	16,850	17,327	13,708	16,084	16,566
Astroid($\gamma = 30$)	22,327	14,679	13,548	28,363	21,073	20,064	14,553	16,860	17,312	13,673	16,082	16,522
Astroid($\gamma = 50$)	22,327	14,679	13,548	28,371	21,189	20,107	14,531	16,838	17,247	13,670	16,060	16,487
Cufflinks	14,977	14,140	13,555	17,700	18,066	17,787	9,082	13,685	14,974	8,761	13,053	14,273
IsoLasso	2,640	5,745	9,237	2,648	5,853	9,814	525	4,405	8,421	232	4,101	8,124
Scripture	53,569	35,114	24,104	56,793	43,334	34,231	7,075	11,416	13,605	N/A	N/A	N/A
Trinity	29,767	30,078	25,193	35,428	38,987	34,349	617	2,390	4,233	N/A	N/A	N/A

Table 2: Summary statistics on the 10M, 20M and 30M 2×75bp paired-end datasets.

Varying fragment length

Table 3 and Table 4 show the summary statistics on two 30M paired-end datasets with mean fragment lengths of 350bp and 450bp, respectively. Combining with the results shown in the main text on the 30M dataset with mean fragment length of 250bp, we see that the performance of Astroid, especially the precision, improves as the the fragment length increases. This demonstrates Astroid's capability of taking advantage of longer fragments to infer the true set of transcripts. Moreover, the correlation computed between the true profiles and the estimated abundance of the matched transcripts of Astroid is much higher than those of Cufflinks and IsoLasso, suggesting that the eTPM estimated by Astroid provides a more accurate quantification of transcript quantities.

Methods	350bp			450bp		
	sensitivity	precision	correlation (long)	sensitivity	precision	correlation (long)
Astroid($\gamma = 0$)	95.23%	88.85%	.923(.919)	95.48%	89.97%	.929(.925)
Astroid($\gamma = 30$)	95.18%	88.60%	.919(.915)	95.47%	89.83%	.927(.923)
Astroid($\gamma = 50$)	95.05%	88.52%	.914(.910)	95.35%	89.69%	.923(.919)
Cufflinks	78.53%	81.97%	0.089(.682)	71.59%	75.57%	.086(.601)
IsoLasso	37.47%	81.04%	.011(.730)	28.95%	68.75%	−0.022(.618)
Scripture	64.97%	34.19%	N/A	58.14%	23.76%	N/A
Trinity	19.61%	9.60%	N/A	11.85%	5.25%	N/A

Table 3: Summary statistics on two 30M 2×75bp paired-end datasets, with mean fragment lengths of 350bp and 450bp respectively.

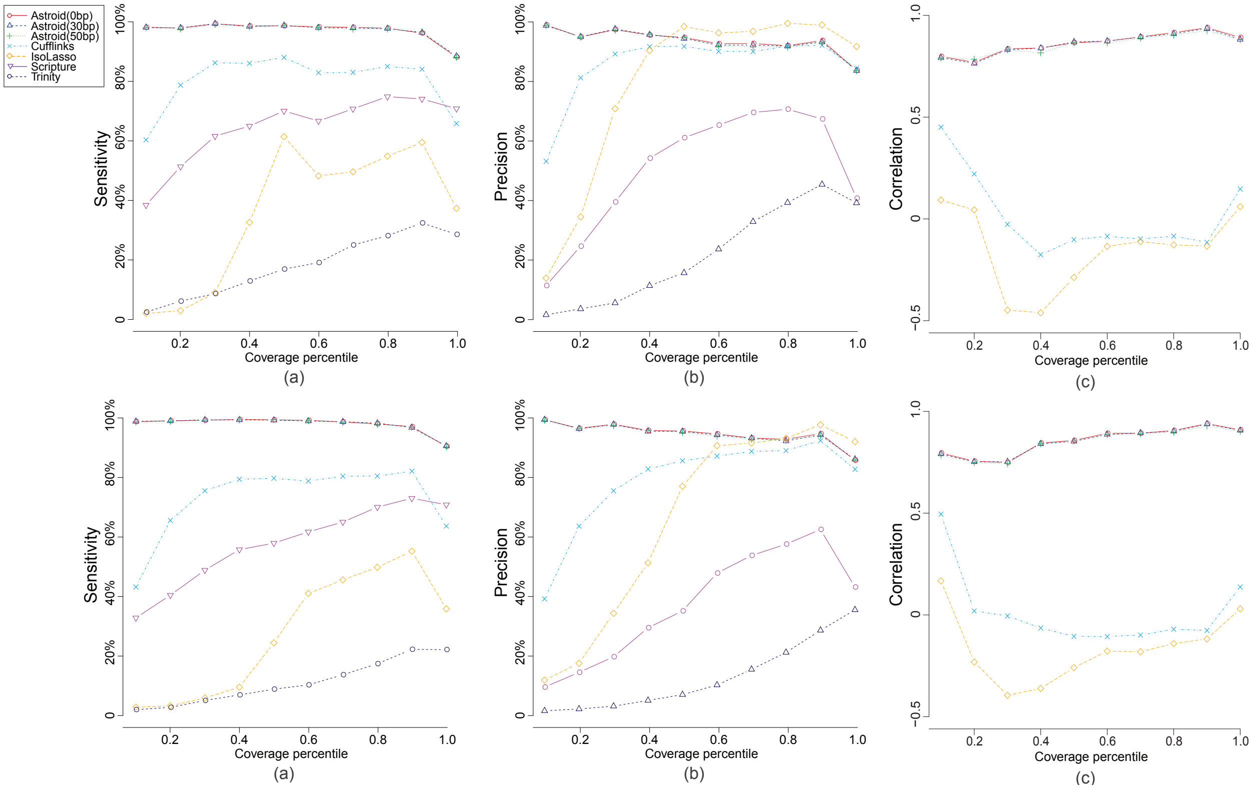

Figure 1: Performance comparison by Astroid with 3 different compression parameters (0bp, 30bp and 50bp), Cufflinks, IsoLasso, Scripture and Trinity on the synthetic human transcriptome dataset. (a), (b) and (c) are the sensitivity, precision and correlation (excluding Scripture and Trinity) against increasing gene coverage when the mean sequenced fragment length is 350bp. (d), (e) and (f) are the sensitivity, precision and correlation (excluding Scripture and Trinity) against increasing gene coverage when the mean sequenced fragment length is 450bp (The legends of these three subfigures are the same as (a), (b) and (c), respectively).

Method	# assembled genes	# assembled transcripts	# matched transcripts	# matched transcripts (long)
	350bp			
Astroid($\gamma = 0$)	13,396	19,693	17,497	16,737
Astroid($\gamma = 30$)	13,396	19,739	17,489	16,740
Astroid($\gamma = 50$)	13,396	19,729	17,465	16,705
Cufflinks	13,553	17,603	14,429	13,725
IsoLasso	8,163	8,496	6,885	6,579
Scripture	26,261	34,869	11,919	N/A
Trinity	28,068	37,552	3,604	N/A
	450bp			
Astroid($\gamma = 0$)	13,385	19,499	17,544	16,783
Astroid($\gamma = 30$)	13,386	19,527	17,541	16,781
Astroid($\gamma = 50$)	13,386	19,533	17,520	16,760
Cufflinks	13,711	17,406	13,154	12,448
IsoLasso	7,520	7,738	5,320	4,993
Scripture	37,320	44,951	10,682	N/A
Trinity	31,332	41,454	2,178	N/A

Table 4: Summary statistics on two 30M 2×75bp paired-end datasets, with mean fragment lengths of 350bp and 450bp respectively, continued.

Time complexity analysis

Table 5 shows the time cost of all the methods on the 30M 2×75bp paired-end read dataset. From this table we can see that by increasing the compression parameter γ, the efficiency is greatly improved for Astroid. Combined with the performance results showed previously, we can see that using the compressed flow network can greatly reduce the time complexity while maintain a good assembly results. In real practice, we can dynamically adjust the compression parameter γ based on the observed read coverage.

Methods	250bp	350bp	450bp
Astroid($\gamma = 0$)	$24h$	$10h$	$4h$
Astroid($\gamma = 30$)	$7h$	$6h$	$1h$
Astroid($\gamma = 50$)	$1h$	$1h$	$40min$
Cufflinks	$40min$	$38min$	$30min$
IsoLasso	$10min$	$5min$	$4min$
Scripture	$15min$	$16min$	$18min$
Trinity	$8h$	$7h$	$6h$

Table 5: Computational performance on there 30M 2×75bp paired-end datasets with different mean fragment lengths. All programs were run on an Intel Xeon E5-2450 32-core 2.10 GHz Linux server with 98GB of RAM.

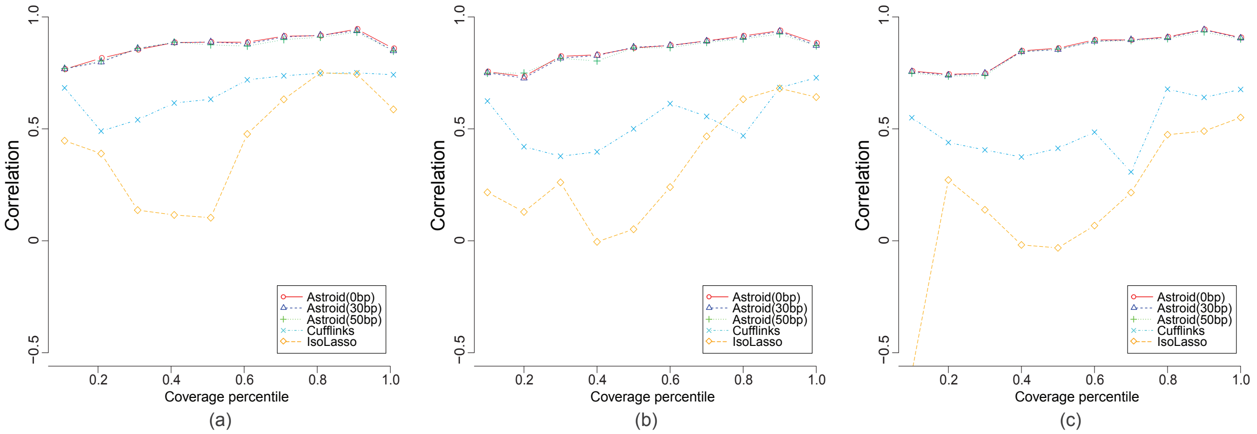

Figure 2: Correlation plot on the subset of matched transcripts with assembled lengths greater than 300bp by each method. (a), (b) and (c) correspond to 30M datasets with different mean sequenced fragment lengths: 250bp, 350bp and 450bp, respectively.
